# Rifampicin/Cotrimoxazole/Isoniazid Versus Mefloquine or Quinine + Sulfadoxine- Pyrimethamine for Malaria: A Randomized Trial

**DOI:** 10.1371/journal.pctr.0010038

**Published:** 2006-12-22

**Authors:** Blaise Genton, Ivo Mueller, Inoni Betuela, Gerard Casey, Meza Ginny, Michael P Alpers, John C Reeder

**Affiliations:** 1 Swiss Tropical Institute, Basel, Switzerland; 2 Papua New Guinea Institute of Medical Research, Goroka, Papua New Guinea

## Abstract

**Objectives:**

Previous studies of a fixed combination including cotrimoxazole, rifampicin, and isoniazid (Cotrifazid) showed efficacy against resistant strains of Plasmodium falciparum in animal models and in small-scale human studies. We conducted a multicentric noninferiority trial to assess the safety and efficacy of Cotrifazid against drug-resistant malaria in Papua New Guinea.

**Design:**

The trial design was open-label, block-randomised, comparative, and multicentric.

**Setting:**

The trial was conducted in four primary care health facilities, two in urban and two in rural areas of Madang and East Sepik Province, Papua New Guinea.

**Participants:**

Patients of all ages with recurrent uncomplicated malaria were included.

**Interventions:**

Patients were randomly assigned to receive Cotrifazid, mefloquine, or the standard treatment of quinine with sulfadoxine–pyrimethamine (SP).

**Outcome Measures:**

Incidence of clinical and laboratory adverse events and rate of clinical and/or parasitological failure at day 14 were recorded.

**Results:**

The safety analysis population included 123 patients assigned to Cotrifazid, 123 to mefloquine, and 123 to quinine + SP. The Cotrifazid group experienced lower overall incidence of adverse events than the other groups. Among the efficacy analysis population (72 Cotrifazid, 71 mefloquine, and 75 quinine + SP), clinical failure rate (symptoms and parasite load) on day 14 was equivalent for the three groups (0% for Cotrifazid and mefloquine; 1% for quinine + SP), but parasitological failure rate (P. falciparum asexual blood-stage) was higher for Cotrifazid than for mefloquine or quinine + SP (9% [PCR corrected 8%] versus 0% and 3%, respectively [*p* = 0.02]).

**Conclusion:**

Despite what appears to be short-term clinical equivalence, the notable parasitological failure at day 14 in both P. falciparum and P. vivax makes Cotrifazid in its current formulation and regimen a poor alternative combination therapy for malaria.

## Editorial Commentary

**Background:** In Papua New Guinea (PNG), malaria is an important cause of death and disease in both adults and children. But concerns exist about whether current antimalarial drugs will be viable for much longer, indicating that new treatments are urgently needed. In 2001, new recommendations on the first-line treatment for uncomplicated malaria were introduced in PNG. These recommendations specify treating older children and adults with the combination of chloroquine together with sulfadoxine–pyrimethamine (SP). However, there is already evidence that malaria parasites in PNG are evolving resistance to this combination therapy. Therefore a group of researchers examined whether new combinations of existing drugs for other diseases could be applied to treatment of malaria in the region. They conducted a trial comparing three different therapies in adults and children over the age of six months who presented to primary care clinics with uncomplicated malaria. The therapies compared were mefloquine (Lariam), quinine taken together with SP, and Cotrifazid, a combination of three different drugs mainly used against tuberculosis. Participants in the trial were followed up for 14 days after treatment, and the main outcome the researchers looked at was treatment failure (i.e., symptoms of clinical malaria together with the presence of malaria parasites in the blood). The researchers also compared the rate of adverse events and presence of malaria parasites in the blood in the different treatment groups.

**What the trial shows:** Clinical treatment failure at day 14 was very low (either 0% or close to 0%) and approximately equivalent in all three treatment groups. The researchers then compared presence of malaria parasites in the blood and found that a much higher proportion of patients treated with Cotrifazid than the other two treatments had parasites in the blood at day 14 (and the difference was statistically significant). Overall the rate of adverse events was lower in the Cotrifazid group than in the other two treatment groups.

**Strengths and limitations:** Studies like this one that examine novel antimalarial treatments are particularly timely, as there is an urgent need to find drugs that will treat malaria resistant to current therapies. In this trial the procedures for randomizing participants to the different treatments were appropriate. However, a key limitation is that patients were followed up for only 14 days, and longer follow-up (as many groups now recommend) might have allowed the researchers to more accurately detect differences in efficacy between the treatments being compared.

**Contribution to the evidence:** Few properly randomized controlled trials have been conducted that look at the ability of Cotrifazid to treat malaria. The results of the trial presented here suggest that Cotrifazid is safe, and short-term clinical efficacy is approximately equivalent to mefloquine or quinine plus SP. However, since in this trial Cotrifazid-treated patients were more likely to have malaria parasites reappear in the blood, Cotrifazid does not seem to be a good alternative treatment in PNG.

## INTRODUCTION

Despite widespread efforts to prevent and treat malaria, worldwide the burden of morbidity and mortality is still high, due in part to the spread of drug-resistant Plasmodium spp. strains. The increasing prevalence of strains of Plasmodium falciparum resistant to 4-aminoquinolines and antifolate drugs has created a crisis in the clinical treatment of malaria in many countries, including Papua New Guinea (PNG) [1]. Introduced in 2001, the new first-line treatment, amodiaquine + sulfadoxine–pyrimethamine (SP) for children weighing less than 20 kilograms or chloroquine + SP for others, is associated with up to 25% failure only two years after its introduction (Marfurt et al., unpublished data). The need is therefore urgent to find and test alternative drugs.

Compounds already on the market for specific indications can easily be screened for effects against neglected diseases. Based on promising preliminary data, a fixed combination including cotrimoxazole, rifampicin, and isoniazid (Cotrifazid) has been developed. It has the advantage of proven safety and tolerability at higher dosage and for longer duration (in tuberculosis).

The rationale to use this combination is based on animal and human experiments. Rifampicin is active against P. berghei malaria in rodents (Brun et al., unpublished data) [2]. In humans, rifampicin showed partial efficacy against P. vivax when associated with primaquine [3]. Numerous studies have shown cotrimoxazole to be active against malaria in humans [4,5]. There is a pharmacokinetic synergism between cotrimoxazole and rifampicin, with increased half-life and AUC (area under the curve) when administered simultaneously [6]. Isoniazid has no clear antiplasmodial activity but delays malaria mortality in mice and reduces overall parasite load when given in combination with rifampicin and cotrimoxazole (Brun et al., unpublished data).

Three studies on Cotrifazid have been conducted in humans in endemic areas, including infants under 6 months of age [7–9]. These studies showed Cotrifazid to be safe and efficacious for the treatment of falciparum malaria, whether uncomplicated, complicated, or drug-resistant [9]. Nevertheless, a formal evaluation was deemed necessary, because the scale of previous studies was small and their methodology was flawed. Additionally, the present study sought efficacy data in geographical areas other than Africa.

The objective of this trial was to compare, in patients with chloroquine- or amodiaquine-resistant malaria, the efficacy and safety of Cotrifazid to that of mefloquine or quinine + SP. The MRAC (Medical Research Advisory Committee) of Papua New Guinea asked that the mefloquine arm be included, to obtain in Papua New Guinean patients reliable safety and efficacy data on alternative drugs that had shown good potential elsewhere.

## METHODS

### Participants

All patients older than 6 months of age presenting at the centres (described below) who were diagnosed with malaria (history of fever, OptiMAL test-positive, no other major symptoms), and who had already been treated for malaria in the 28 days before, qualified for inclusion in the study if the individual or legal guardian (for children) gave informed consent and if the clinician in charge would have given the standard treatment for drug-resistant malaria independent of the study. A participant was excluded if the clinician preferred to use quinine + SP for any reason (in case of “moderately severe” malaria), or if the patient had one of the symptoms or signs of complicated or severe malaria (i.e., history of recent convulsion, any neurological sign or impairment of consciousness, heavy vomiting, haemoglobinuria, respiratory distress, bleeding, circulatory collapse, shock, jaundice, or haemoglobin [Hb] <5 g/dl), had contraindications for mefloquine (history of psychiatric disorder or epilepsy), or was pregnant.

All patients suspected on clinical grounds to have drug-resistant malaria were investigated by a nurse employed specifically for the trial who performed the OptiMAL test (Diamed, Cressier, Switzerland). If the latter was positive (any *Plasmodium* species), patients were screened for other inclusion and exclusion criteria and recruited when appropriate.

The patients were recruited in four primary care health facilities: the outpatient clinic of Maprik hospital serving Maprik District, the Kunjingini health subcentre serving the Wosera area, the outpatient clinic of Yagaum hospital serving the Amele community near Madang, and the Madang outpatient clinic serving Madang town and its surroundings. The Maprik and Wosera areas are situated in the East Sepik Province and the Madang area is in the Madang Province, PNG. Malaria transmission is intense and perennial in both provinces, the Wosera being the most endemic with an overall P. falciparum prevalence in the general population of 60% [10,11]; in the Madang area, the falciparum prevalence is about 40% [12,13].

### Design

This was an open-label, block-randomised, comparative, multicentric study. Patients were randomly assigned to receive oral Cotrifazid, mefloquine, or quinine + SP.

### Interventions

Cotrifazid, a fixed combination of rifampicin 112.5 mg, sulfamethoxazole 200 mg + trimethoprim 40 mg, and isoniazid 75 mg (Fatol Arzneimittel GmbH, Schiffweiler, Germany), was supplied as a coated tablet that could be stored at room temperature. The treatment dosage and schedule for patients was as follows. Patients weighing 40 kg or more: two tablets twice per day; patients under 40 kg and 20 kg or more: one tablets twice per day; patients under 20 kg: one-half tablet twice day. These doses were given every 12 hours for 7 days (days 0–6).

The treatment regimen for mefloquine (Lariam tablet containing mefloquine 250 mg [F. Hoffmann-La-Roche Ltd, Basel, Switzerland]) was given at the usual dosage of 25 mg/kg in children, 1,250 mg for adults less than 60 kg, and 1,500 mg for adults over 60 kg, split in two doses at hours 0 and 12.

Quinine (a commercial product in PNG) was given as usual: 10 mg/kg every 8 hours for 5 days (days 0–4). In this regimen, patients also received a single dose of 0.5–3 tablets of Fansidar (containing 500 mg of sulfadoxine and 25 mg of pyrimethamine [F. Hoffmann-La-Roche Ltd, Basel, Switzerland]) on day 0.

The doses in the morning of days 0–3 were administered under the supervision of the appointed nurses at the study centres. They were responsible for confirming both the participant identification number and the label of the trial drugs. Intake of the afternoon doses on day 3 was checked by interview on day 4, and intake of doses on days 4–6 by interview on day 7.

Follow-up assessments were done on days 1, 2, and 3 (clinical and parasitological), 7 (clinical, parasitological, and biochemical), and 14 (clinical, parasitological, and haematological), or more intensively in individual cases of persisting symptoms or pathological signs. Blood samples were taken by venipuncture (2 ml) on days 0 and 7 and by fingerpick on days 1, 2, 3, and 14. For details on assessment procedures, see “Laboratory Procedures” below.

Treatment was changed to quinine + SP if the patient failed treatment with other drugs. There was no rescue treatment if the failure occurred after quinine + SP; a second course with the same drugs was to be given, as stated in the national guidelines at the time of the study.

### Objectives

The specific objectives of the trial were (i) to compare the efficacy of Cotrifazid to that of the standard treatment for drug-resistant malaria in PNG (quinine + SP) and to another drug (mefloquine) that is being considered for future use, and (ii) to compare the tolerance of Cotrifazid with that of quinine + SP and mefloquine in the same population. The trial was designed to test the null hypothesis that the clinical cure rate with Cotrifazid is not inferior to that with the comparators (mefloquine or quinine + SP).

### Outcomes

#### Safety parameters.

The prime measurement of safety was incidence of clinical or laboratory adverse events (AEs). All patients were followed clinically every day for the first four days, and longer in cases of complication. All patients were seen on days 7 and 14 to identify late AEs or clinical failure. Laboratory measurements included serum glutamic-oxaloacetic transaminase (aspartate aminotransferase) (SGOT), serum glutamate-pyruvate transaminase (alanine aminotransferase) (SGPT), and creatinine on days 0 and 7, and Hb concentration on days 0 and 14.

AEs were defined according to standard criteria, i.e., any adverse change from the participant's baseline (pretreatment) condition (including clinically relevant laboratory abnormalities, abnormal physical signs, and intercurrent illnesses), irrespective of whether the event was considered related to the trial drug or not. The occurrence of serious AEs, as defined by standard criteria, was recorded and acted upon. There was no assessment of AE intensity or relatedness to the product investigated because of the confounding effect of malaria symptoms and signs.

#### Efficacy parameters.

The primary parameter of efficacy was the clinical treatment failure rate on day 14, using a blood slide as gold standard for parasitology. Clinical treatment failure was defined as (i) the occurrence of severe malaria between day 1 and day 14, or (ii) the persistence or recurrence of symptoms or signs (including temperature >37.5 °C) associated with any parasitaemia between day 5 and day 14.

Secondary parameters of efficacy were (i) parasitological failure rate on day 14, (ii) fever clearance time, (iii) parasite clearance time, (iv) symptom clearance time, (v) occurrence of complications (information collected on days 1, 2, 3, 7 and 14) and Hb concentration (from samples collected on day 14).

### Sample Size

The overall sample size of 330 (110 in the Cotrifazid group, 110 in mefloquine, and 110 in quinine + SP treatment) was chosen to test the hypothesis that Cotrifazid was not inferior to mefloquine or quinine + SP, assuming a rate of treatment success of 95% with mefloquine or quinine + SP and a clinically acceptable rate of treatment success of 86% or more in the Cotrifazid group, and a 10% loss to follow-up (80% power, 95% confidence limits, one-sided test) [14].

### Randomisation—Sequence Generation

Assignment to treatment groups was done by a randomisation list (block of 12, i.e., four Cotrifazid, four mefloquine, and four quinine + SP), which had been computer-generated (SAS Software) by a statistician of the Swiss Tropical Institute not involved in the study. The original list was kept at the Swiss Tropical Institute and a copy at the PNG Institute of Medical Research headquarters.

### Randomisation—Allocation Concealment

Sequential numerical codes (1–390 to accommodate potential errors at inclusion) were written on the reverse side of sealed envelopes that had been prepared at the Swiss Tropical Institute and forwarded to the local investigator prior to the start of the study. Inside the envelope, the treatment group was concealed on a paper with either the letter “L” for Lariam (mefloquine), “C” for Cotrifazid, or “S” for standard (quinine + SP) written on it.

### Randomisation—Implementation

Once a patient had met the inclusion criteria and his or her (or the guardian's) consent had been given, the patient was assigned the code number following the one of the previous patient. The envelope corresponding to that code number was opened by the research nurse, and the first dose of the allocated treatment administered under supervision. The entire process was to be completed before any procedure was started for the following patient.

### Blinding

This was an open-labelled trial. Neither the research nurse nor the patients were blinded to the treatment given.

### Laboratory Procedures

#### Parasitology.

The OptiMAL test was used at screening (day 0) to document malaria.

On days 0, 1, 2, 3, 7, and 14 malarial parasites were assessed by microscopy. Thick and thin films were stained with Giemsa at pH 7.2. Before a slide was declared negative 100 thick film fields were examined by microscopy. The number of malaria parasites per 200 white blood cells was counted. The number of asexual forms per μl was then calculated using a mean white blood cell count of 10,000/μl in children below the age of 5 y and of 8,000/μl in persons aged 5 y or older. The standard quality control performed at the PNG Institute of Medical Research was applied [11].

#### Haematology.

Hb concentration was determined using a photometer (Hemocue, Hemocue Ltd, Sweden).

#### Biochemistry.

SGOT, SGPT, and creatinine were measured with an automated dry chemical photometer (Reflotron System, Boehringer-Mannheim, Mannheim, Germany). Regular quality controls were run for all the measurements.

#### Parasite genotyping.

P. falciparum genotype profiles were assessed on samples of day 0 and day of failure using a combination of several molecular markers of the SP resistance genes *dhfr* and *dhps*.

### Ethics

The trial was approved by the Medical Research Advisory Committee of PNG. Verbal informed consent from each participant in the trial or from his/her parents or guardian(s) was obtained in front of a witness after explanation of the aims, methods, benefits, and potential hazards of the trial.

### Monitoring

A trial monitor (Isi Kevau, University of Papua New Guinea, Port Moresby, Papua New Guinea) reviewed all procedures and ensured complete adherence to the protocol.

### Statistical Methods

#### Statistical methods.

Data were double-entered using a specific program written in FoxPro software version 3.0 and analysed using Stata software version 8.2.

#### Safety.

All participants who received at least one daily dose of Cotrifazid or comparators and who presented to one follow-up visit were included in the safety analysis population. Comparisons of the incidence of AEs (following prompted questions) reported at follow-up contacts (days 1–14) were performed using a Poisson model. Axillary temperatures, respiratory rates, and Hb values are reported as mean ± standard deviation (SD).

#### Efficacy.

All participants who showed asexual parasites (any species) at baseline and who received the treatment with Cotrifazid, mefloquine, or quinine + SP for at least the first three days (days 0–2), and who presented to the follow-up visit on day 14, were included in the per protocol efficacy analysis population. Efficacy was estimated by comparing the proportions of complications and the rates of clinical and parasitological failures between the Cotrifazid and comparator groups, using the Mantel-Haenzel Chi-square test with α = 0.05, one-tailed) or Fisher's exact test where appropriate. A Kaplan-Meier survival analysis of all asexual parasites, fever, and symptoms was performed, and treatment groups compared using the log rank test. The Student's t-test was used to compare means of Hb concentration between the groups.

An intention-to-treat analysis was also performed to assess parasitological outcomes at day 14, counting all children who didn't appear for assessment on that day as failures.

## RESULTS

### Participant Flow and Number Analysed

**Figure 1:**
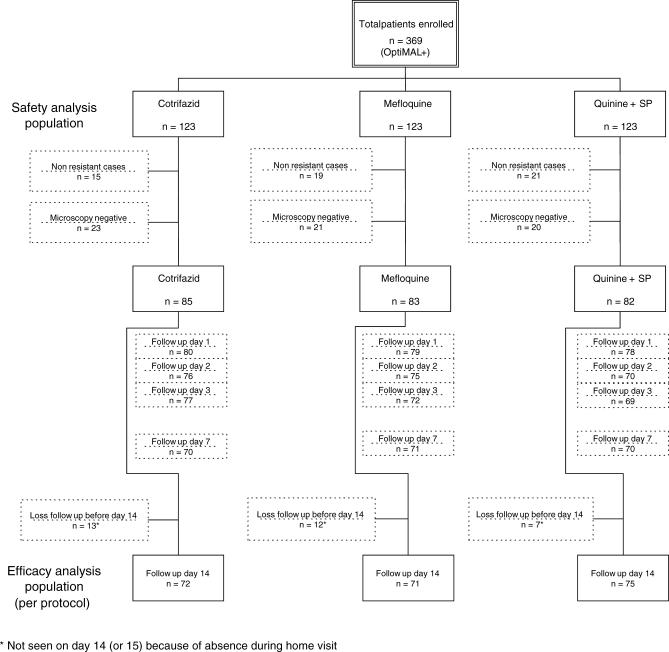
Participant Flow

The safety analysis population included 137 patients in the Maprik hospital outpatient clinic, 49 in Kunjingini health subcentre, and 183 in Madang town outpatient clinic. No patient was admitted to the ward. The participant flow is detailed in Figure 1. A total of 369 patients were included, using the OptiMAL test to detect parasitaemia: 123 in the Cotrifazid group, 123 in the mefloquine group, and 123 in the quinine + SP group. Of the total in this group, 55 were excluded on day 2 since they were found not to have been treated with antimalarials in the last 28 days after reviewing their health book (nonresistant malaria). Another 64 were excluded because the microscopical investigation did not confirm the presence of asexual blood-stage parasites. The lower detection of microscopy when compared to OptiMAL is most likely due to the detection of circulating gametocytes by the rapid test, a phenomenon that was not documented when the study was designed and conducted.

In the efficacy analysis population for the outcome measures (clinical and parasitological failures on day 14), 32 patients were lost to follow-up on day 14, which left 218 patients (72 Cotrifazid, 71 mefloquine, and 75 quinine + SP) (see Figure 1).

### Recruitment

The study was conducted from April 2000 to January 2003.

### Baseline Data

The female:male ratio was 180:184 (five were unknown). In the Cotrifazid group the median age was 7.2 y (range 1.4–45 y), mefloquine 7.4 y (range 0.5–56 y), and quinine + SP 8 y (range 0.5–61 y).

The prevalence of reported symptoms and observed signs at baseline for each treatment group is described in Table 1. More patients had subjective and objective fever in the quinine + SP group: 45% (55/123) had a temperature over 37.5 °C at baseline versus 32% (39/123) for Cotrifazid and 34% (42/123) for mefloquine. Haematology values and number of patients with abnormal liver and kidney function tests at baseline are shown in Table 1. Elevated values of SGOT were found in 26% of the Cotrifazid and quinine + SP patients and 20% of the mefloquine patients.

**Table 1:**
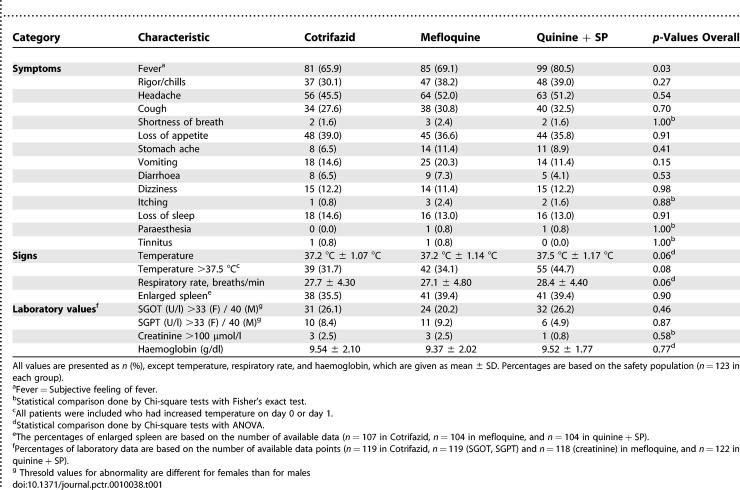
Summary of Symptoms, Signs, and Laboratory Values by Treatment Group at Baseline

Parasitology at baseline showed that 94% (80/85) of the patients were infected with P. falciparum in the Cotrifazid group, 90% (75/83) in the mefloquine group, and 87% (71/82) in the quinine + SP group. The corresponding values for P. vivax were 8.2% (7/85), 9.6% (8/83), and 23% (19/83). A higher prevalence of mixed infections was found in the quinine + SP group (10% versus 4% in the Cotrifazid group and 4% in the mefloquine group). Geometric mean densities were equivalent.

Full compliance with the treatment evaluated from direct observation in the first three days and history-taking thereafter was 93% (115/123) in the Cotrifazid group (total of 7 d), 98% (121/123) in the mefloquine group (2 d), and 96% (118/123) in the quinine + SP group (5 d). Two patients interrupted their treatment due to AEs, both in the quinine + SP group; both were cured although none of them took an alternative drug.

### Outcome and Estimation: Adverse Events

No deaths occurred in this study. Among the 369 patients included in the safety analysis, only one patient suffered from a serious AE. This 1.7-y-old child, who was enrolled in the Cotrifazid group, required hospital admission due to the persistence of high fever, cough, and vomiting on day 3. The child had 32,640 parasites/μl on day 0, 40 parasites/μl on day 2, and none from day 3 onwards. Thus, the fever was very likely of nonmalarial origin. The child was put on standard treatment with quinine + SP and antibiotics, and made a full recovery. One child in the Cotrifazid and two in the mefloquine group experienced an impairment of their level of consciousness, but none was severe enough to warrant rescue treatment with quinine + SP or hospital admission. One additional child developed chest indrawing during the follow-up in the Cotrifazid group. In the Cotrifazid group, four participants had yellow eye colour during follow-up (days 1, 2, 3, and 7), but none in the other groups. The few other non-prompted AEs—weakness in four patients, red urine in three (all in the Cotrifazid group), sneezing in three, facial swelling in one, and swollen lymph nodes in one—were considered isolated events, and none required the administration of ancillary treatment.

The Cotrifazid group experienced a significantly lower overall incidence of prompted AEs during follow-up than the other groups. Table 2 details the treatment-specific incidence of these AEs recorded on days 1, 2, 3, 7, or 14, without distinction between those related to the drug or to the malaria episode. Vomiting was reported significantly less often in the Cotrifazid group than in the mefloquine group (*p* < 0.001); stomach ache (*p* < 0.05), vomiting (*p* < 0.01), dizziness (*p* < 0.05), and tinnitus (*p* < 0.01) were less common in the Cotrifazid than in the quinine + SP group. In contrast, participants in the Cotrifazid group were reported shortness of breath significantly more often than did those in the mefloquine group (*p* < 0.01) (see Table 2 for details).

**Table 2:**
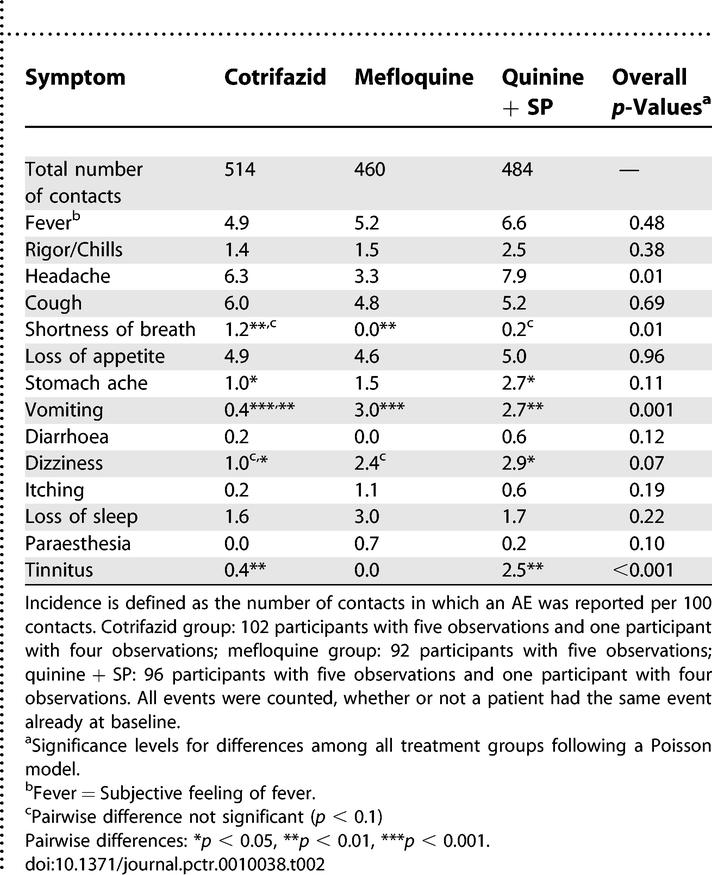
Summary of Incidences of AEs at Follow-Up (Days 1, 2, 3, 7, and 14) per 100 Contacts

All abnormal biochemical tests observed at baseline (Table 1) had resolved by day 7, which was the only day of laboratory follow-up for these values.

### Outcome and Estimation: Efficacy

#### Clinical failure.

Among the 218 patients retained in the efficacy analysis population, no patients developed signs of severe malaria based on the clinician's judgment, nor did any require secondary hospital admission due to malaria. No patients who were initially treated with Cotrifazid or mefloquine required rescue treatment with quinine + SP. No clinical treatment failures occurred with Cotrifazid or mefloquine, but one late treatment failure (patient was asymptomatic but had a temperature of 37.7 °C and parasite density of 80/μl on day 14) occurred in the quinine + SP group. The three treatment groups were therefore equivalent with regard to the clinical cure rate (100% in Cotrifazid and mefloquine groups and 99% in quinine + SP group, *p* = 0.32, Fisher's exact test). However, due to the low sample size finally included in the efficacy analysis population, we had only 80% power to find a 4-fold increase in failure rate, assuming a failure rate of 5% in the quinine + SP group.

#### Parasitological failures.

Parasitology results at baseline and on day 1, 2, 3, 7, and 14 (per protocol analysis) are detailed in Table 3.

**Table 3:**
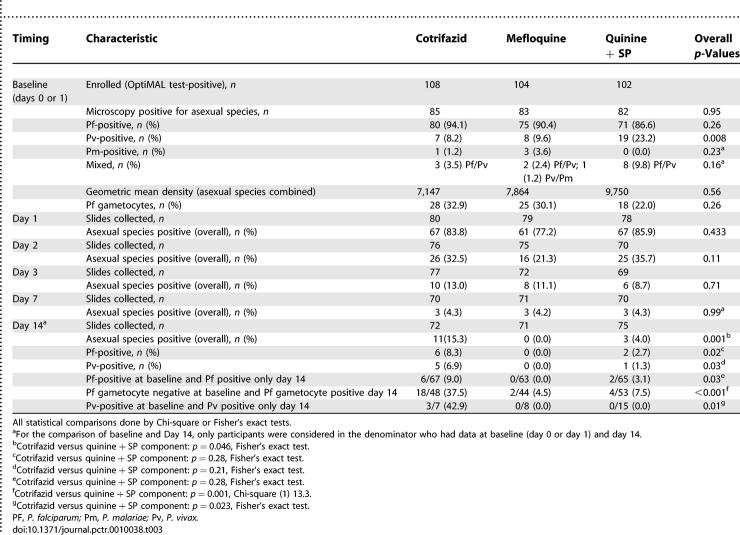
Summary of Parasitological Results at Enrolment and during Follow-Up (Days 1, 2, 3, 7 and 14): Per Protocol Analysis

When all patients sampled on a given day were taken into account (irrespective of the other time points and species at baseline), there was no difference in parasite clearance on day 1, 2, 3 and 7 (4% positive in all groups for the latter). However, on day 14, the prevalence of asexual blood-stage *Plasmodium*-positive patients was significantly higher in the Cotrifazid group at 15% (11/72) than in the mefloquine group, 0% (0/71), and the quinine + SP group at 4% (3/75) (Fisher's exact test, *p* = 0.001). The rates for P. falciparum on day 14 were 8% (6/72), 0% (0/71), and 3% (2/75) respectively (*p* = 0.02, Fisher's exact test).

When patients with P. falciparum only at baseline and during follow-up were considered, 9% (6/67) had asexual blood-stage parasitaemia on day 14 in the Cotrifazid group versus 0% (0/63) in the mefloquine group and 3% (2/65) in the quinine + SP group (Fisher's exact test, *p* = 0.03). This difference was not significant when the Cotrifazid group was compared to the quinine + SP group (Table 3). Parasite genotyping on individuals with reappearing P. falciparum parasites showed that only 13% (1/8) of the failures were due to new infections, which gives a PCR-corrected failure rate of 8% (versus 9% uncorrected). Patients in the Cotrifazid group were more likely to develop P. falciparum gametocytaemia on day 14 than those in the mefloquine or quinine + SP groups: 38% (18/48) versus 5% (2/44) and 8% (4/53), respectively (*p* < 0.001, Fisher's exact test) (Table 3).

When patients with P. vivax only at baseline and during follow-up were considered, the parasite prevalence on day 14 was 43% (3/7) in the Cotrifazid group, 0% (0/8) in the mefloquine group, and 0% (0/15) in the quinine + SP group (*p* = 0.01, Fisher's exact test).

Intention-to-treat analysis did not lead to major changes (see Table 4 for details). In fact, the prevalence of asexual blood stage–positive patients (counting the missing ones as positive) at day 14 was still significantly higher in the Cotrifazid group (28%) than in the mefloquine group (15%) or the quinine + SP group (12%) (*p* < 0.001, Fisher's exact test). The rates for P. falciparum were 22%, 15%, and 11% respectively, and those for P. vivax 21%, 15%, and 10% (Table 4).

**Table 4:**
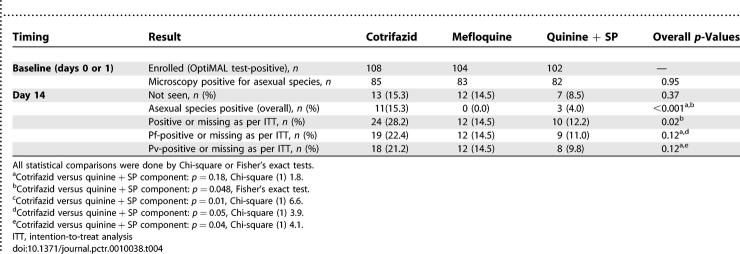
Summary of Parasitological Results at Enrollment and Day 14: Intention-to-Treat Analysis

#### Survival analysis.

The survival analyses are based only on complete sequences of follow-up up to event occurrence.

#### Fever clearance time.

**Figure 2:**
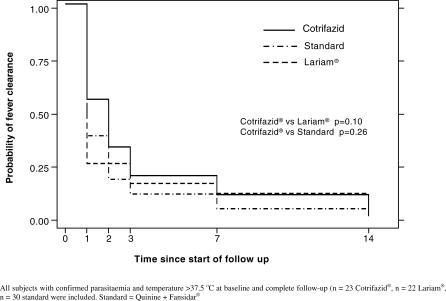
Time to Fever Clearance: Kaplan-Meier Analysis

Figure 2 shows the fever clearance time in the three treatment groups. There was no significant difference between the groups treated with Cotrifazid, mefloquine, or quinine + SP.

#### Parasite clearance time.

**Figure 3:**
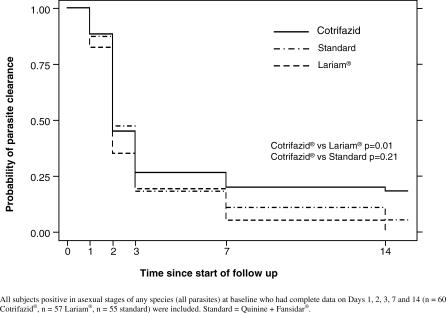
Time to Clearance of All Asexual Parasites: Kaplan-Meier Analysis

Figure 3 shows that the clearance of all asexual parasites in the Cotrifazid group was slower than in the other treatment groups, the difference with the mefloquine group being significant (*p* = 0.01). There was also a tendency for a slower clearance of P. falciparum in the Cotrifazid group as compared to the other groups, but the differences did not reach statistical significance.

#### Haemoglobin concentration.

The mean Hb concentrations on day 14 were increased compared to the pretreatment values on day 0: 10.4 ± 1.9 (post-treatment) versus 9.54 ± 2.1 g/dl (Cotrifazid group), 10.4 ± 2.2 (post-treatment) versus 9.37 ± 2.0 g/dl (mefloquine group), and 10.2 ± 1.9 (post-treatment) versus 9.52 ± 1.8 g/dl (quinine + SP group). There was no statistical difference between groups on day 0 (*p* = 0.77) or on day 14 (*p* = 0.73).

## DISCUSSION

### Interpretation

The present study shows that Cotrifazid, a combination of rifampicin, sulfamethoxazole, trimethoprim and isoniazid, is safe and efficacious for the alleviation of symptoms and signs of drug-resistant malaria (clinical failure rate of 0% for Cotrifazid and mefloquine, and 1% for quinine + SP), but is insufficient to clear all parasites, especially those of P. vivax in semi-immune Papua New Guinean populations.

#### Safety.

The overall incidence of prompted AEs in the Cotrifazid group was lower than that observed in the mefloquine and quinine + SP groups. The types of AEs recorded in these last two groups were consistent with those of the literature, which also validates the data recorded for Cotrifazid. Except for “shortness of breath,” none of the prompted AEs was more frequent in the Cotrifazid group (Table 2). The precise relatedness of the AEs to the drugs used is not reported, since most AEs observed are also those that are encountered during a malaria episode. The known clinical and laboratory AEs of the components included in the Cotrifazid combination were not observed, except for the red-coloured urine—an AE that is not medically deleterious.

#### Efficacy.

Although the final sample size for efficacy analysis was smaller than expected because the OptiMAL test used for screening was positive in the presence of gametocytes only, we were able to demonstrate equivalence of the three regimens in curing uncomplicated malaria (primary outcome). Indeed, all treatment groups had almost 0% clinical failure, a rate that was lower than the 5% expected in the sample size calculation. The short duration of our follow-up is likely to have impacted positively on treatment outcome, although it is not different from the one used in several recent trials. A follow-up of 28–42 d, as proposed by White [15] for long-acting drugs, would certainly have allowed us to detect additional clinical failures, especially in the Cotrifazid group in which 9% of the patients infected with P. falciparum at baseline had recurrent parasites by microscopy on day 14. At the time of study design (1999), we decided on a 14-day follow-up since it was, and is still now, the standard duration chosen to determine policy change in areas of intense transmission [16]. Despite the short follow-up, we are still able to compare our results with recent trials testing other new drugs or regimens in Africa that also used 14 days for assessing treatment outcome [17–24].

The situation is less promising when considering parasitological failures. Of the patients treated with Cotrifazid, 15% were parasitaemic (any *Plasmodium* species) on day 14 versus 4% for quinine + SP and 0% for mefloquine. These results reflect slower and less effective parasite clearance (Figure 3) after Cotrifazid treatment, especially for P. vivax. Most of the reappearing P. falciparum parasites (87%) were true recrudescence, as expected with a follow-up of 14 days, as well as from results of previous studies done in PNG [25,26] and recent data from in vivo studies conducted in the same area, where we found that only 6% (4/72) of the reappearing parasites on day 14 were due to new infections (Marfurt et al., unpublished data). The situation is less clear for *P. vivax,* but a recurrence is more likely than a new infection or relapse in a time period of 14 days. Circulating asexual stages of P. vivax after blood schizonticidal therapy might originate from asexual parasites that survived therapy, from activated hypnozoites that led to a relapse, or from a new infection. Unlike with P. falciparum infections, in which true recrudescence can be distinguished from new infections by the use of genotyping methods, current molecular methods used for the genetic analysis of P. vivax do not allow the unambiguous classification of recurrent parasitaemia, and hence of true treatment failure.

### Generalizability

The observation of no clinical treatment failure for Cotrifazid but a significant rate of parasitological failure is a matter of great concern. In endemic areas, parasitological failure is usually the first sign of reduced activity of the drug used; adequate clinical responses are still seen, despite persistent parasitaemia, because host immunity helps to alleviate symptoms [15]. The early parasitological failure rate, the low efficacy to suppress P. falciparum gametocytogenesis, and the rather long regimen are the main reasons to conclude that Cotrifazid is not an appropriate combination therapy, especially in areas where both P. falciparum and P. vivax infections coexist.

### Overall Evidence

Three studies were conducted with Cotrifazid in the past, all of rather small scale and one with serious methodological flaws (the randomisation was stopped at an early stage due to the refusal of patients to be given the standard treatment because of the perceived lower rate of adverse events and excellent efficacy of Cotrifazid) [7–9]. The present clinical trial allowed Cotrifazid to be assessed in a large sample size of patients, in an area where different *Plasmodium* species coexist, and with effective drug regimens used as comparators. Our results agree with previous ones, namely good safety profile and excellent efficacy to alleviate symptoms. However, Cotrifazid in our study was slightly less effective in clearing P. falciparum parasites than in the African studies (8% failure versus 5% respectively) [9]. Although small, this difference might be due to the fact that parasites from PNG have responded to different and more intense selective pressures than those in Africa, and resemble more the Southeast Asian parasites with a multidrug resistance profile. This might be especially so because we recruited only patients with 4-aminoquinoline-resistant malaria; in the African study, this group had 33% (2/6) parasitological failure. The conclusion of the African study, “Cotrifazid is very well suited for the treatment of malaria tropica, also in cases of apparent drug resistance of P. falciparum against other antimalarials, and even in severe cases of the disease” [9], does not, in our opinion, accurately reflect their results, and is therefore overoptimistic, especially in view of the tendency to only consider a treatment acceptable if the overall failure is less than 10%.

We believe that the design and power of our trial was optimized to accurately assess Cotrifazid safety and efficacy, and the results therefore should robustly support any conclusions on the usefulness of Cotrifazid as an antimalarial drug. Despite its very good safety profile, short-term clinical equivalence with effective drugs such as mefloquine, and low cost, Cotrifazid for malaria does not appear to be an ideal alternative therapeutic option in its current formulation and regimen: patients experience a slower parasite clearance, some recurrence of asexual forms, and higher gametocytaemia on day 14 than after mefloquine or quinine + SP treatment. However, in formulating new and affordable combinations of antimalarial drugs in the future, the clinical efficacy of the Cotrifazid components could be taken into account, especially if it is considered an advantage, as part of the Integrated Management of Childhood Illness strategy [27], to use drugs that may act on both malaria and pneumonia.

## SUPPORTING INFORMATION

CONSORT Checklist Click here for additional data file. (45 KB DOC)

Trial Protocol Click here for additional data file. (133 KB DOC)

## Abbreviations

AE: adverse event
Hb: haemoglobin
PNG: Papua New Guinea
SD: standard deviation
SGOT: serum glutamic-oxaloacetic transaminase (aspartate aminotransferase)
SGPT: serum glutamate-pyruvate transaminase (alanine aminotransferase)
SP: sulfadoxine–pyrimethamine
