# Supplementary material for: Rifampicin/Cotrimoxazole/Isoniazid Versus Mefloquine or Quinine + Sulfadoxine- Pyrimethamine for Malaria: A Randomized Trial
Source: PLoS Clin Trials. 2006 Dec 22;1(8):e38. doi: 10.1371/journal.pctr.0010038 (PMC1713262; doi:10.1371/journal.pctr.0010038)
Supplement: Trial Protocol [file pctr.0010038.sd002.doc]

**CLINICAL TRIAL PROTOCOL**

**Efficacy and tolerance of COTRIFAZID (rifampicin, co-trimoxazole, isoniazid) against resistant malaria in semi-immune subjects:**

**a multicentric, open, block-randomised, comparative trial**

Papua New Guinea Institute of Medical Research, Maprik, Papua New Guinea

Swiss Tropical Institute, Basle, Switzerland

24 April 1999

**Date of protocol**: April 2 4, 1999

**Protocol title**: Efficacy and tolerance of COTRIFAZID (rifampicin, co-trimoxazole, isoniazid) against resistant malaria in semi-immune subjects: a multicentric, open, block-randomised, comparative trial

**Version**: fourth draft

Number of centres: 2

**INVESTIGATORS**

Principal Investigator: Blaise Genton MD PhD MSc DTM&H

Clinical epidemiologist, Department of Medical Parasitology and Infection Biology

Swiss Tropical Institute

Socinstrasse 57

Postfach

4002 Basel Switzerland

Telephone number: + 41 61284 81 30

Fax number: + 41 61 271 86 54

Email : Blaise.genton@chuv.hospvd.ch

Co-principal investigator : Inoni Betuela MD

Papua New Guinea Institute of Medical Research

Po Box 400

Maprik Papua New Guinea

Telephone number: + 675 858 14 14

Fax number: + 675 858 12 57

Email : imrmap@datec.com.pg

PROTOCOL TITLE Efficacy and tolerance of COTRIFAZID (rifampicin, co- trimoxazole, isoniazid) against resistant malaria in semi-immune subjects: a multicentric, open, block- randomised, comparative trial

PROJECT PHASE III

SPONSOR Fatol Arzneimittel GmbH

INDICATION UNDER STUDY Resistant malaria

OBJECTIVES 1) To compare the efficacy of Cotrifazid versus Lariam® (mefloquine) or standard treatment in semi-immune subjects with resistant malaria

2) To compare the tolerance of Cotrifazid versus Lariam® or standard treatment in semi-immune subjects with resistant malaria

EXPERIMENTAL DESIGN

OF THE TRIAL Open, block-randomised, comparative

PLANNED SAMPLE SIZE 330 (110 Cotrifazid, 110 Lariam®, 110  standard

treatment)

NUMBER OF CENTRES 2

SUBJECT SELECTION CRITERIA Subjects with resistant malaria (any species) with a weight > 5kg

SUBJECT EXCLUSION CRITERIA Pregnant women, infants less than 6 months of age, severe malaria

TREATMENT Cotrifazid (rifampicin, co-trimoxazole, isoniazid)

DOSAGE Subjects > 40 kg : 2 x 2 tablets per day for 7 days

Subjects < 40 kg and > 20kg : 2 x 1 tablet per day for 7d

Subjects < 20 kg : 2 x ½ tablet  per day for 7 d

SCHEDULE Every 12 hours

ROUTE OF ADMINISTRATION Oral

COMPARATOR I Lariam® (mefloquine)

DOSAGE Subjects > 60 kg : 6 tabs (3, 2, 1)

Subjects > 40 kg and < 60 kg : 5 tabs (2, 2, 1)

Subjects < 40 kg and > 30 kg : 3 ½ tabs (1 ½ , 1, 1)

Subjects < 30 and > 20 kg : 3 tabs (1, 1, 1)

Subjects < 20 kg and > 10 kg : 1 ½ tabs ( ½ , ½, ½ )

Subjects < 10 kg : ¾ tablet ( ¼ , ¼, ¼ )

SCHEDULE Hour 0, 8, 24

ROUTE OF ADMINISTRATION Oral

COMPARATOR II Standard treatment (presently quinine + Fansidar®)

DOSAGE Quinine

Subjects > 60 kg : 3 x 600 mg /day for 5 days Subjects < 60 kg : 3 x 10 mg/kg/day for 5 days

+

Fansidar® as a single dose

Subjects > 40 kg : 3 tablets

Subjects < 40 kg and > 30 kg : 2 tablets

Subjects < 30 and > 20 kg 1 ½ tablet

Subjects < 20 kg and > 10 kg 1 tablet

Subjects < 10 kg ½ tablet

SCHEDULE Every 8 hours for quinine, hour 0 for Fansidar®

ROUTE OF ADMINISTRATION Oral for quinine and Fansidar®, or I.M. for quinine

Parameters OF EFFICACY 1. Fever clearance time

1. Parasite clearance time
2. Rate of early and late treatment failure (ETF, LTF)
3. Occurrence of complications

PARAMETERS OF TOLERANCE: 1. Reported Adverse Events (AEs)

2. Clinical and laboratory values

Secondary Parameters of EfficaCY 1. Symptom clearance time

2. Haemoglobin concentration

PROCEDURES

All subjects who are diagnosed with malaria (history of fever and no other major symptom, OptiMAL test positive) and have been already treated for malaria in the last 28 days can be included if they give their informed consent and if the clinician in charge would have given the standard treatment for resistant malaria in the absence of the study. A patient should not be included if the clinician prefers to use quinine for whatever reason, if the patient has one of the symptoms or signs of complicated or severe malaria(i.e. history of recent convulsion, any neurological sign or impairment of consciousness, temperature >40 C^0^, heavy vomiting, haemoglobinuria, respiratory distress, bleeding, circulatory collapse, shock, jaundice, haemoglobin < 5 g/dl), is less than 6 months of age or is pregnant. The patients will be randomised on the spot to have either Cotrifazid, Lariam® or standard treatment (at present quinine + Fansidar®). The treatment dosage and schedule for Cotrifazid will be the following : subjects > 40 kg : 2 x 2 tablets, subjects < 40 kg and > 20kg : 2 x 1 tablet, subjects < 20 kg : 2 x ½ tablet, ever 12 hours for 7 days. The treatment dosage and schedule for Lariam® will be the following : subjects > 60 kg : 6 tabs (3 at hour 0, 2 at hour 8, 1 at hour 24), subjects > 40 kg and < 60 kg : 5 tabs (2, 2, 1), subjects < 40 kg and > 30 kg : 3 ½ tabs (1 ½ , 1, 1), subjects < 30 and > 20 kg : 3 tabs (1, 1, 1), subjects < 20 kg and > 10 kg : 1 ½ tabs ( ½ , ½, ½ ), subjects < 10 kg : ¾ tablet ( ¼ , ¼, ¼ ). Follow-up assessments will be done at hours 24, 48 and 72 (clinical, parasitological, pharmacological) and days 7 (clinical, parasitological, biochemical and pharmacological) and 14 (clinical, parasitological, haematological, pharmacological), or more intensively in case of persisting symptoms or pathological signs. Treatment will be changed to the standard treatment (at present quinine + Fansidar®) if necessary (problems of tolerance, aggravation of clinical status, increase in parasitaemia etc with Cotrifazid or Lariam®).

STATISTICAL ANALYSIS

EFFICACY :

1. Comparison of the fever clearance time in the Cotrifazid and comparator groups
2. Comparison of the parasite clearance time in the Cotrifazid and comparator groups
3. Comparison of the rate of ETF and LTF in the Cotrifazid and comparator groups
4. Comparison of the rate of complications in the Cotrifazid and comparator groups

5. Comparison of the haematological concentration in the Cotrifazid and comparator groups

TOLERANCE:

1. Comparison of the rate of adverse events in the Cotrifazid and comparator groups
2. Comparison of the type of adverse events in the Cotrifazid and comparator groups
3. Comparison of the rate of patients requiring an additional drug because of tolerance problems in the Cotrifazid and comparator groups (e.g. antivomiting)
4. Comparison of the biochemical and haematological results in the Cotrifazid and comparator groups

**TABLE OF CONTENTS Page**

**1 JUSTIFICATION AND OBJECTIVES**

1.1 Background and Rationale. 8

1.2 Objectives of the Trial. 10

**2 GENERAL DESIGN**

2.1 Overall Design. 10

2.2 Number of Centres. 11

**3 SUBJECT SELECTION FOR STUDY ENTRY**

3.1 Population Base. 11

3.2 Inclusion Criteria. 11

3.3 Exclusion Criteria. 11

3.4 Subject Screening. 11

**4 DESCRIPTION OF TRIAL DRUG**

4.1 Names and Formulations. 12

4.2 Packaging, Storage and Blinding. 12

**5 STUDY PROCEDURES**

5.1 Drug 12

5.1.1Drug Administration 12

5.1.1.1 Dosage Regimen & Rationale 12

for Selection.

5.1.1.2 Route of Administration. 13

5.1.1.3 Duration. 13

5.1.1.4 Dispensing & Accountability of Trial 13

Drug Supplies.

5.1.2 Assignment to Drug or Comparator Groups. 13

5.1.3 Concomitant Medication. 13

5.2 Assessment of Safety. 13

5.2.1 Safety Parameters (incl. Laboratory Evaluations). 13

5.2.2 Adverse Events. 14

5.2.3 Immediately Reportable Adverse Events. 16

1. Drug administration and Follow-up of Adverse Events. 17

5.3 Assessment of Efficacy 17

5.3.1 Primary Efficacy Parameters 17

1. Secondary Efficacy Parameters 17

5.4 Assessment of pharmacology 17

**6 WITHDRAWAL**

6.1 Conditions for Withdrawal. 17

6.2 Replacement Policy. 18

**7 ETHICS**

7.1 Declaration of Helsinki and Good Clinical Practice. 18

7.2 Informed Consent. 18

7.3 Ethics Committee Approval. 18

7.4 Confidentiality and Data Protection. 18

**8 ADMINISTRATIVE PRACTICALITIES**

8.1 Drug Supply 19

8.2 Monitoring. 19

8.3 Sponsor’s Responsibilities. 19

8.4 Investigator's Responsibilities. 20

8.5 Publications. 21

**9** **SUBJECT ANALYSIS POPULATIONS** 21

**10 STATISTICAL STATEMENT**

10.1 Statistical Hypothesis. 21

10.2 Study Design. 21

10.3 Sample Size Calculation. 21

10.4 Strategy for Statistical Analysis. 21

1. Efficacy Parameters 21
2. Primary Efficacy Parameters 22
3. Secondary Efficacy Parameters 22

10.4.2 Primary Safety Parameters 22

10.5 Data Management and Analysis. 22

**11 REFERENCES** 22

**12 SIGNATURE PAGE** 24

**1 JUSTIFICATION AND OBJECTIVES**

**1.1 Background and Rationale**

Malaria remains a serious health problem in most developing countries, with approx. 2.5 million deaths per year. One of the contributor to mortality is the spreading of malaria resistance first in Southeast Asia and then in Africa and the South Pacific. In Papua New Guinea (PNG), chloroquine-resistant falciparum malaria was first documented in 1976 (Yung and Bennett, 1976; Grimmond *et al.*, 1976). Since then, studies done in different provinces at different times showed the problem to be widespread and to increase gradually with a recent shift from RI to RII and RIII types (Dulay *et al.*, 1987; Schuurkamp, 1992; Trenholme *et al.*, 1993; Al-Yaman *et al.*, 1996). In the Wosera, Genton *et al*. (unpublished data,1998) showed that 16 to 26% of the patients who were treated with the standard regimen for uncomplicated malaria (amodiaquine or chloroquine) had a recrudescence of symptoms with parasitaemia.

Sulfadoxine/pyrimethamine (Fansidar®)-resistant falciparum malaria was described in 1980 (Darlow *et al.*, 1980; Darlow *et al.*, 1982). As far as quinine is concerned, the first documented cases of in vivo resistance of *P. falciparum* in PNG were described in 1993 (Al-Yaman *et al.*, 1993), with a clear increase since then, in spite of a prolonged duration of treatment (5 days instead if 3).

Because of the above failures, the PNG medical authorities have planned to introduce gradually a new artemisinine derivative to replace quinine and Fansidar®, which is the present standard regimen for complicated or resistant malaria. At present, the main problem is not the lack of potent drugs, but the lack of potent and cheap drugs. Indeed, the combination of atovaquone and proguanil (Malarone), or more recently the combination of benflumetol and artemether (Riamet) have proved to be safe and efficacious, but they are unaffordable for people living in endemic areas. Mefloquine (Lariam®) has proved to be quite efficacious for a while in South East Asia against multiresistant falciparum malaria, and also reasonably cheap. In the last decade, treatment failures have become more frequent and artemeter was added in combination with mefloquine to the standard treatment regimen for falciparum malaria in multidrug resistant areas of Thailand (Price et al, 1995). It is possible that a fixed combination (artemisinine derivative + mefloquine) will be available in the future to fight multiresistant parasites in highly endemic areas. In this context, testing mefloquine alone in PNG is a step towards the development of safe and efficacious drugs and drug combinations for the country when quinine and Fansidar® will not be sufficient anymore.

In the same sense, the development of Cotrifazid is promising. Indeed it meets most of the requirements for a new drug for resistant malaria, which are the following : it is a combination made of relatively short-acting compounds (decreasing the rapid spread of resistant strains) ; it is active against resistant strains of *Plasmodium falciparum* in vitro and it is efficacious in mice infected with resistant strains (Brun *et al*., unpublished data) ; it is likely to be safe since the compounds have been used either alone or in combination for a very long time for the treatment of tuberculosis for example, with higher dosage for a much longer duration ; it is likely to be cheap (approx. 5 dollars for a full treatment).

There have been some studies conducted with Cotrifazid in humans living in endemic areas, including infants of less than 6 months of age (Freerksen *et al.*, 1995,1996, Goerg *et al.*, in press). All these studies showed Cotrifazid to be safe and efficacious for the treatment of falciparum malaria, whether uncomplicated, complicated or resistant (Goerg *et al*, in press). It is required to have additional hard data in terms of efficacy against resistant malaria, and also to know about efficacy in geographical areas other than Africa.

The rationale to use this combination is based on animal and human experiments. Rifampicin is active against *Plasmodium berghei* malaria in rodents (Alger et al, 1970 ; Brun, unpublished data). Numerous studies showed co-trimoxazole to be efficacious in the treatment of malaria in humans (Bloland et al, 1991 ; Hansford et al, 1982 ; Hutchinson & Farquhar, 1982). There is a pharmacokinetic synergism between co-trimoxazole and rifampicin, with increase of t_50_ and AUC when administered simultaneously (Bhatia et al, 1991). Isoniazid has no clear anti-plasmodial activity. It has only been shown to delay malaria mortality in mice, and to reduce overall parasite load when given in combination with rifampicin and co-trimoxazole (Brun, unpublished data). This may be due to an effect of isoniazid on blood cytokines, in particular TNF-alpha, since isoniazid did protect mice against endotoxin lethality (Urbaschek et al, 1991). The last argument to include isoniazid in the combination is to avoid the selection of resistant mycobacteria when treating for malaria an undiagnosed tuberculous patient. This risk has been revisited recently, and clear-cut evidence was presented that such a event was extremely improbable (Mueller-Brundaler et al, 1997). Even WHO, when defining a case of ‘previously untreated’ pulmonary tuberculosis for the purpose of an eventual ‘primary drug resistance’ identification, accepts for such a patient a previous antituberculous chemotherapy of up to 1 month duration (WHO, 1997). This is based also on the experience that, even with rifampicin monotherapy, the probability of selecting for rifampicin resistance of tubercle bacilli is very low. Moreover, should one fear the possibility of a not recognized advanced pulmonary tuberculosis case receiving Cotrifazid for malaria, then isoniazid as the usual combination drug for treatment of that disease would offer an additional protection against the selection of rifampicin-resistant bacilli.

Study area

Within the Malaria Vaccine Epidemiology and Evaluation Project (MVEEP) situated in the Wosera in Maprik District, East Sepik Province, and in Yagaum, Madang Province (Alpers *et al.*, 1992), we have conducted baseline studies of the epidemiology and transmission of malaria, specific parasitological and immunological studies, as well as clinical and drug resistance studies (see Cattani et al 1986, Cox et al 1994, Genton *et al.*, 1994a,b, 1995, 1997). Infrastructure including research offices and laboratories, as well as accommodation are in place. These facilities are all within 5 minutes walk of the outpatient clinics and hospitals where the study will take place.

**1.2 Objectives of the Trial**

The purpose of this trial is to assess the efficacy and safety (primary parameters) of Cotrifazid in semi-immune Papua New Guinean patients with resistant malaria.

More specifically i) to compare the efficacy of Cotrifazid versus Lariam® (mefloquine) or standard treatment in semi-immune subjects with resistant malaria, and ii) to compare the tolerance of Cotrifazid versus Lariam® or standard treatment in semi-immune subjects with resistant malaria.

**2 GENERAL DESIGN**

**2.1 Overall Design**

This is an open, block-randomised, comparative, multicentric study.

All subjects presenting at the outpatient clinic or admitted in the ward of the Maprik Hospital and Yagaum Health Center who are diagnosed with malaria (history of fever and no other major symptom, OptiMAL test positive) and have been already treated for malaria in the last 28 days can be included if they give their informed consent and if the clinician in charge would have given the standard treatment for resistant malaria in the absence of the study. A patient should not be included if the clinician prefers to use quinine for whatever reason, if the patient has one of the symptoms or signs of complicated or severe malaria (i.e. history of recent convulsion, any neurological sign or impairment of consciousness, temperature >40 C^0^, heavy vomiting, haemoglobinuria, respiratory distress, bleeding, circulatory collapse, shock, jaundice, haemoglobin < 5 g/dl), has contra-indications for mefloquine (history of psychiatric disorder, epilepsy), is less than 6 months of age, or is pregnant. The patients will be randomised on the spot to have either Cotrifazid, Lariam® or standard treatment (at present quinine + Fansidar®). The treatment dosage and schedule for Cotrifazid will be the following : subjects > 40 kg : 2 x 2 tablets, subjects < 40 kg and > 20kg : 2 x 1 tablet, subjects < 20 kg : 2 x ½ tablet, ever 12 hours for 7 days. The treatment dosage and schedule for Lariam® will be the following : subjects > 60 kg : 6 tabs (3 at hour 0, 2 at hour 8, 1 at hour 24), subjects > 40 kg and < 60 kg : 5 tabs (2, 2, 1), subjects < 40 kg and > 30 kg : 3 ½ tabs (1 ½ , 1, 1), subjects < 30 and > 20 kg : 3 tabs (1, 1, 1), subjects < 20 kg and > 10 kg : 1 ½ tabs ( ½ , ½, ½ ), subjects < 10 kg : ¾ tablet ( ¼ , ¼, ¼ ). Follow-up assessments will be done at hours 24, 48 and 72 (clinical, parasitological and pharmacological), and days 7 (clinical, parasitological, biochemical, and pharmacological) and 14 (clinical, parasitological, haematological, and pharmacological), or more intensively in case of persisting symptoms or pathological signs. Treatment will be changed to the standard treatment (at present quinine + Fansidar®) if necessary (problems of tolerance, aggravation of clinical status, increase in parasitaemia etc).

All blood samples will be taken by venepuncture (2 ml).

**2.2 Number of Centres**

This is a multiple centre study which will take place at the Maprik Hospital, East Sepik Province, and at Yagaum Health Center, Madang Province, Papua New Guinea.

**3 SUBJECT SELECTION FOR STUDY ENTRY**

**3.1 Population Base**

Subjects will be selected from people continuously exposed to malaria, living in the catchment area of the Maprik Hospital and Yagaum Health Center.

**3.2 Inclusion Criteria**

Willingness of the adults, parents or legal guardian for children, to give informed consent is the primary criteria for inclusion.

Other inclusion criteria will be : persons presenting at the outpatient clinic or admitted in the ward, aged > 6 months, with fever or history of fever in the last three days, and no other major symptom or sign, with a clinical diagnosis of malaria, an OptiMAL test positive (for whatever species), and with a documented (in the health book) episode of malaria treated with standard drugs in the last 28 days.

**3.3 Exclusion Criteria**

A subject is not eligible if one or more of the following parameters are fulfilled:

- History of allergy to drug compounds
- Unable to swallow
- Signs of severe malaria (i.e. history of recent convulsion, any neurological sign or impairment of consciousness, temperature >40 C^0^, heavy vomiting, , haemoglobinuria, respiratory distress, bleeding, circulatory collapse, shock, jaundice, haemoglobin < 5 g/dl)
- Contra-indications for Lariam® (history of psychiatric disorder, epilepsy)
- Less than 6 months of age
- Pregnancy

**3.4 Subject Screening**

During the screening phase, subjects are to be identified by their name.

A screening log must be kept by the investigator, in which the eligibility status of **every** subject screened must be recorded, regardless of whether or not they enter the study. To maintain confidentiality, subjects need only be identified by their initials.

**4 DESCRIPTION OF TRIAL DRUG**

The drug is produced by Fatol Arzneimittel GmbH.

**4.1 Names and Formulations**

Cotrifazid is a combination made of rifampicin 112.5 mg, sulphamethoxazole 200 mg + trimethoprim 40 mg, and isoniazid 75 mg.

**Galenical Form and Strength.**

Cotrifazid is a coated tablet.

**4.2** **Packaging, Storage and Blinding**

The drug is stored at room temperature.

Investigators and subjects are aware of the treatment allocation, i.e. Cotrifazid, Lariam® or standard treatment.

**5** **STUDY PROCEDURES**

**5.1 Drug**

**5.1.1 Drug Administration**

**5.1.1.1 Dosage Regimen & Rationale for Selection**

The treatment dosage and schedule for Cotrifazid will be the following : subjects > 40 kg 2 x 2 tablets, subjects < 40 kg and > 20kg  2 x 1 tablet, subjects < 20 kg  2 x ½ tablet, every 12 hours.

The treatment dosage and schedule for Lariam® will be the following : subjects > 60 kg : 6 tabs (3 at hour 0, 2 at hour 8, 1 at hour 24), subjects > 40 kg and < 60 kg : 5 tabs (2, 2, 1), subjects < 40 kg and > 30 kg : 3 ½ tabs (1 ½ , 1, 1), subjects < 30 and > 20 kg : 3 tabs (1, 1, 1), subjects < 20 kg and > 10 kg : 1 ½ tabs ( ½ , ½, ½ ), subjects < 10 kg : ¾ tablet ( ¼ , ¼, ¼ ).

The treatment dosage and schedule for quinine will be the following : subjects > 60 kg  3 x 600 mg quinine /day, subjects < 60 kg  3 x 10 mg/kg/day every 8 hours.

The treatment dosage and schedule for Fansidar® will be the following : subjects > 40 kg  3 tablets, subjects < 40 kg and > 30 kg 2 tablets, subjects < 30 and > 20 kg 1 ½ tablet, subjects < 20 kg and > 10 kg 1 tablet, subjects < 10 kg ½ tablet.

**5.1.1.2 Route of Administration**

Cotrifazid, Lariam® and Fansidar® will be given orally.

Quinine will be given orally or intramuscularly..

**5.1.1.3 Duration**

Cotrifazid will be given for 7 days.

Lariam® will be given for 24 hours.

Quinine will be given for 5 days.

Fansidar® will be given as a single dose at day 0.

**5.1.1.4 Dispensing of Trial Drug**

The administration of the drug for the first three days will be supervised by the nurse in charge of the trial.

All drug supplies are to be returned to Fatol Arzneimittel GmbH.

**5.1.2 Assignment to Drug or Comparator Groups**

An independent person will generate a randomisation list from a computer program with treatment allocation by block of 12 ; he will assign a unique code number for each person (1-330). The treatment allocation will be written, and kept in an envelope that will be opened once the subject has given his/her consent.

The time of treatment administration will be written on the case report form.

**5.1.3. Concomitant Medication**

In the present trial the only medications that cannot to be used are the ones that have an antimalarial activity, i.e. amodiaquine, chloroquine and tetracyclines.

**5.2 Assessment of Safety**

**5.2.1 Safety Parameters (including Laboratory Evaluations)**

The prime measurement of safety in this trial is the close observation of the subjects. All subjects will be followed clinically every day for the first 4 days, and longer in case of complication. All subjects will be seen at day 7 and 14 to identify late adverse events or clinical failure.

Laboratory measurements : AST and ALT will be measured at Day 0 and 7, haemoglobin concentration at day 14.

## 5.2.2 Adverse Events

An adverse event is **ANY** type of adverse change from the trial subject's pre-drug baseline condition. **ALL** adverse events MUST be reported by the investigator without 'filtration', whether considered treatment-related or not. The wording used in English will be drawn from a list of preferred terms, with their closest equivalent in pidgin.

## In this study AEs will be measured against the medical history, baseline physical examination and laboratory baseline values.

## AEs should be described by severity, intensity and relationship to the compounds under study as well as the method by which the information was obtained (observed, elicited or spontaneously reported).

The severity/intensity of adverse events is graded on a 3-point scale as follows:

**MILD:** Discomfort noted, but no disruption to normal daily activities.

**MODERATE:** Discomfort sufficient to reduce or affect normal daily activities.

**SEVERE:** Inability to perform normal daily activities.

**All** adverse events encountered during the clinical trial will be reported in the case report form.

An **adverse event** is defined as any adverse change from the subject's baseline pre-treatment condition (signs and symptoms at baseline), which occurs after chemotherapy has started, whether considered related or not to the drug. This includes clinically relevant laboratory abnormalities and intercurrent illnesses which occur or worsen during the study period.

The intensity of adverse events will be graded on a three-point scale (mild, moderate and severe) and described in detail, along with the investigator's assessment of the relationship of the event to treatment.

The investigator's assessment of the event's relationship to the trial treatment should be defined according to the following standard statement:

**Categories for Determining Relationship to Trial Drug:**

**PROBABLE** (must have first three)

This category applies to those adverse events which are considered, with a high degree of certainty, to be related to the test drug. An adverse event may be considered **probable**, if:

1. It follows a reasonable temporal sequence from administration of the drug.

2. It cannot be reasonably explained by the known characteristics of the subject's clinical state, environmental or toxic factors, or other modes of therapy administered to the subject.

3. It disappears or decreases on cessation or reduction in dose. (There are important exceptions when an adverse event does not disappear upon discontinuation of the drug, yet drug-relatedness clearly exists; eg. [1] bone marrow depression, [2] tardive dyskinesias.)

4. It follows a known pattern of response to the suspected drug.

5. It reappears upon re-challenge.

**POSSIBLE** (must have the first two)

This category applies to those adverse events in which the connection with the test drug administration appears unlikely, but cannot be ruled out with certainty. An adverse event may be considered **possible** if, or when:

1. It follows a reasonable temporal sequence from administration of the drug.

2. It may have been produced by the subject's clinical state, environmental or toxic factors, or other modes of therapy administered to the subject.

3. It follows a known pattern of response to the suspected drug.

**REMOTE** (must have the first two)

In general, this category is applicable to an adverse event which meets the following criteria:

1. It does **not** follow a reasonable temporal sequence from administration of the drug.

2. It may readily have been produced by the subject's clinical state, environmental or toxic factors, or other modes of therapy administered to the subject.

3. It does not follow a known pattern of response to the suspected drug.

4. It does not reappear or worsen when the drug is re-administered.

**UNRELATED**

This category is applicable to those adverse events which are judged to be clearly and incontrovertibly due only to extraneous causes (disease, environment, etc.) and do not meet the criteria for drug relationship listed under REMOTE, POSSIBLE or PROBABLE.

**5.2.3 Immediately Reportable Adverse Events**

Any adverse event that is considered SERIOUS (as defined below) must be reported IMMEDIATELY by the investigator, ie. within one working day of becoming aware of the event.

An **Immediately Reportable Adverse Event (IRAE)** is any serious adverse event or abnormal laboratory test value that occurs during the defined treatment period, and which suggests a significant hazard, contraindication, side effect or precaution. An IRAE **must** be immediately reported to the clinical monitors **within one working day**.

***Immediately Reportable adverse events*** include any event or experience that is:

- fatal;

- life threatening;

- permanently disabling (ie. severely incapacitating or interfering with the ability to resume usual life patterns);

- requires inpatient hospitalisation or prolonged hospitalisation;

- an overdose (ie. a deliberate or inadvertent administration of a treatment at a dose higher than that specified in the protocol *and* higher than known therapeutic doses for that specific indication);

The definition of an IRAE includes any event which is expected or unexpected, related or unrelated to the drug.

All IRAEs must also be reported on the adverse events page of the CRF and must be assessed for severity and the relationship to the trial medication. The actions taken by the investigator and the outcome of the event must be reported.

**IRAEs must be reported to the appropriate ethics committee, if requested by the committee and/or according to local legal requirements.**

It is important that the SEVERITY of an adverse event is not confused with the SERIOUSNESS of the event. For example, vomiting which persists for many hours may be severe, but is not necessarily a serious adverse event. On the other hand, stroke which results in only a limited degree of disability may be considered a mild stroke, but would be a serious adverse event.

**5.2.4 Drug Administration and Follow-Up of Adverse Events**

All adverse events MUST be documented and followed up until the event is either resolved or adequately explained, even after the subject has completed their trial treatment.

**5.3 Assessment of Efficacy**

**5.3.1 Primary Efficacy Parameters**

The primary parameters of efficacy i) fever clearance time, ii) parasite clearance time, iii) failure rate, iv) occurrence of complications, information collected from day 1,2,3, 7 and 14.

Thick and thin blood films will be Giemsa stained and species-specific density will be counted per/200 white blood cells (WBC). Slides will be declared negative if no parasites are seen in 100 thick film fields. Parasite density per microliter blood will be calculated by multiplying the parasitemia/200 WBC by a WBC count of 10000/μL in children aged < 5 years and of 8000/μL in persons aged > 5 years.

1. **Secondary Efficacy Parameters**

The secondary parameters of efficacy are i) symptom clearance time, information collected at day 1,2,3, 7 and 14, and ii) haemoglobin concentration from samples collected at day 14.

1. **Assessment of Pharmacology**

Pharmacokinetics will be assessed in a small subsample of patients (5). Blood will be drawn (2 ml by venepuncture) at hour 2, 8, 16, 24, 36, 72.

**6 WITHDRAWAL**

**6.1 Conditions for Withdrawal**

1. Failure of the subject to attend scheduled visits.
2. Adverse events (including intercurrent illnesses).
3. Occurrence of complications - if Cotrifazid or Lariam®, change to standard treatment, if quinine + Fansidar®, persist with standard treatment (including other available drugs as considered clinically appropriate)
4. Violations of, and deviations from, the protocol.
5. Subject withdraws consent.
6. Subject lost to follow-up.
7. Administrative/other.

***Subjects may withdraw from the trial at any time and for any reason, without affecting their right to treatment by the investigator****.* The investigator has the right to withdraw a subject for any reason which is in the best interests of the subject, including intercurrent illness, adverse events, or treatment failure.

Although withdrawals should be avoided if at all possible, it is understood that withdrawals may occur during a trial. Whenever a subject is withdrawn from a trial, FOR WHATEVER REASON, a final trial evaluation must be completed for that subject, stating the reason for withdrawal. All documentation concerning the subject must be as complete as possible.

Withdrawals due to non-attendance must be followed up by the investigator to obtain the reason for non-attendance.

WITHDRAWALS DUE TO INTERCURRENT ILLNESS OR ADVERSE EVENTS MUST BE FULLY DOCUMENTED IN THE CASE RECORD FORM, WITH SUPPLEMENTARY INFORMATION WHERE AVAILABLE AND/OR APPROPRIATE.

**6.2 Replacement Policy**

Recruitment of replacement subjects is unnecessary.

**7 ETHICS**

The responsibility for conducting clinical trials to the highest possible standards lies with the sponsor, monitor, and investigator.

**7.1 Declaration of Helsinki and Good Clinical Practice**

The investigator will ensure that this trial is conducted in full conformance with the principles of the *Declaration of Helsinki* (as amended in Tokyo, Venice and Hong Kong).

Furthermore, it is the responsibility of the investigator to ensure that the trial is performed in accordance with Good Clinical Practice (cf Klinische Arzneimittelprüfungen in der EU), and with all local laws and regulations concerning clinical trials.

This protocol complies with the Department of Health and Human Services (HHS) regulations for the protection of Human Research Subjects (45 CRF 46).

**7.2 Informed Consent**

The investigator will obtain verbal informed consent from each subject participating in the trial or from his/her parents or guardians, after explanation of the aims, methods, benefits and potential hazards of the trial (written informed consent is inappropriate in this setting where few parents are able to write). The consent will be obtained in front of a witness and BEFORE any trial-specific procedures will be performed on the subject.

It will be made completely and unambiguously clear to each subject that they are free to refuse to participate in the trial, or to withdraw their consent at any time and for any reason, without incurring any penalty or withholding of treatment on the part of the investigator.

**7.3 Ethics Committee Approval**

It is the responsibility of the investigator(s) to submit a copy of this protocol and consent form to the Medical Research Advisory Committee of Papua New Guinea in order to obtain INDEPENDENT approval to conduct the trial. Approval from the committee must be obtained before starting the study, and should be documented in a letter to the investigator specifying the date on which the committee met and granted the approval

**7.4 Confidentiality and Data Protection**

The investigator must ensure that the subject's anonymity will be maintained. Subjects will be identified on the case report forms by their ID. The investigator will keep a separate confidential enrolment log which matches identifying codes with the subjects' names and residencies.

**8 ADMINISTRATIVE PROCEDURES**

**8.1 Drug Supply**

Fatol Arzneitmittel GmbH will supply the investigator with the drug and sufficient support to conduct the trial according to the agreed protocol.

**8.2 Monitoring**

Fatol Arzneitmittel GmbH will provide a trained “Trial Monitor” to assist the investigator in conducting the trial. This monitor will have the responsibility of reviewing the ongoing trial with the investigator to verify adherence to the protocol and to deal with any problems if and when they arise. At all times the monitor will maintain the confidentiality of trial documents.

**8.3 Sponsor’s Responsibilities**

A special insurance will be contracted to (i) provide treatment and/or compensation in the event of an injury to or the death of a subject, as a result of his/her participation in this clinical trial, and (ii) insure the investigator(s) against or indemnify them for losses resulting from liability for such injuries or deaths.

**8.4 Investigator's Responsibilities**

The investigator agrees to conduct the trial in accordance with the procedures and requirements laid out in this protocol. In particular, the investigator agrees to conduct the trial in accordance with Good Clinical Practice and strict ethical principles (see section 7). Any modification to the agreed protocol must be approved, if appropriate, by the ethics committee approving the original protocol, BEFORE any modifications are put into effect.

It is the responsibility of the investigator to complete and sign a case report form for each subject in the trial.

Each case report form must be completed legibly. Erroneous values and/or text must not be obliterated (for example, covered with correction fluid). Instead, the error should be crossed out with a single line, the correct value/text added, and the correction signed and dated by the investigator.

In the event of an expected or unexpected serious adverse event (IRAE), the investigator is to notify the clinical monitors within one working day. IRAEs are to be reported, whether or not they are considered related to the treatment.

In addition to the case report form, the investigator will maintain adequate records that fully document the progress of the trial. Copies of these trial records and related documents must be kept on file by the investigator for a period of no less than five years. ALL DOCUMENTATION AND MATERIAL FOR THIS TRIAL (CASE REPORT FORMS, PROTOCOL, ETC.) ARE TO BE RETAINED IN A SECURE PLACE AND TREATED AS CONFIDENTIAL MATERIAL.

The investigator agrees to allow the monitors to access to any or all of the trial materials needed for source data verification and proper review of trial progress. The investigator (or deputy) agrees to assist the monitor in resolving any problems that may be detected during or after the study period.

The investigator agrees to comply with regulatory authority requirements regarding the auditing of this trial.

The investigator has the right to request premature termination of the trial for administrative or other reasons. Should this be necessary and agreed upon, the necessary procedures will be arranged after review and consultation by all parties to ensure protection of the subjects' interests.

By signing this document the investigator indicates that he/she has read and fully understood the protocol, and agrees to abide by all the requirements of the protocol.

**8.5 Publications**

One of the goals of a clinical trial, in addition to demonstrating safety and efficacy, is to provide publications.

For this study all papers should be authored by representatives of PNGIMR and STI.

**9 SUBJECT ANALYSIS POPULATIONS**

All subjects who are randomised to receive either Cotrifazid or the comparators constitute the ‘*intention to treat population’*.

Subjects will be excluded from analysis of *safety* if they do not receive at least one daily dose of Cotrifazid or comparators, and one follow-up visit. Remaining subjects constitute the ‘*safety population’.*

Subjects will be excluded from final analysis of *efficacy* if they do not receive the full dose of Cotrifazid, or the full dose of Fansidar® + at least 3 daily dose of quinine, and one follow-up visit. Remaining subjects constitute the ‘*efficacy population’*.

**10 STATISTICAL STATEMENT**

**10.1 Statistical Hypothesis**

Study hypotheses:

i) Cure rate of resistant malaria after Cotrifazid treatment is not inferior to the one obtained after treatment with mefloquine or quinine and Fansidar®.

ii) Cotrifazid is safe to use in semi-immune patients with resistant malaria. No IRAE attributable to the drug is expected in the subjects.

**10.2 Study Design**

This is a multicentric, open, block-randomised, comparative study.

1. **Sample Size Calculation**

The overall sample size of 330 (110 in Cotrifazid group, 110 in Lariam® group and 110 in standard treatment group) is sufficient to demonstrate that Cotrifazid is not inferior to Lariam® or quinine and Fansidar®, assuming a rate of treatment success of 95% with Lariam or quinine and Fansidar® and a clinically acceptable rate of treatment success of 86% or more in the Cotrifazid group, a 10% loss of follow-up (80% power, 95% confidence limits, one-sided test) (formula from Jones et al, 1996).

**10.4 Strategy for Statistical Analysis**

**10.4.1 Efficacy Parameters**

**10.4.1.1 Primary Efficacy Parameters**

Efficacy will be estimated by comparing the rate of treatment failure, and proportions of complications between the Cotrifazid and comparator groups, using the Mantel-Haenzel Chi-square test (x^2^=0.05, one-tailed). The time to fever clearance and the time to parasite clearance will also be compared between the Cotrifazid and comparator groups. Survival curves showing the clearance time will be plotted for the two groups, and compared using the logrank test.

**10.4.1.2 Secondary Efficacy Parameters**

Efficacy will be estimated by comparing the time to symptom clearance, and haemoglobin concentration at day 14 between the Cotrifazid and comparator groups. Survival curves showing the clearance time will be plotted for the two groups, and compared using the logrank test. The t test will be used to compare means of haemoglobin concentration between the two groups.

**10.4.2 Primary Safety Parameters**

Rates of acute and subacute AEs by severity and relation to the drug will be compared between the Cotrifazid and the comparator groups. Laboratory parameters (haematological) in the two groups will be compared.

The Mantel-Haenzel Chi-square test (x^2^=0.05, two-tailed) will be used to assess any significant difference between the two groups in terms of number of AEs reported.

**10.5 Data Management and Analysis**

Data cleaning and entry will be done in Madang, under the supervision of I. Betuela and B. Genton.

Weekly and monthly backups on tapes, the latter being kept in Maprik and Madang will be made.

Data analysis will be done by I. Betuela, and B. Genton (PNGIMR, STI).

Discussion of analysis will involve all collaborating persons.

All papers will include representatives of PNGIMR and STI.

**11 REFERENCES**

Alger NE et al., *Nature* 1970 ;227 :361-362.

Alpers M et al., *Papua New Guinea Med J* 1992;35:285‑297.

Al‑Yaman F et al., *Papua New Guinea Med J* 1994; 37: 54-56.

Al-Yaman F et al., *Papua New Guinea Med J* 1996; 39: 16-22.

Bhatia RS et al., *Hum Exper Toxicol*  1991 ;10 :419-421.

Bloland PB et al., *Lancet* 1991 ; 337: 518-520.

Cattani JA et al., *Am J Trop Med Hyg* 1986; 35**:** 3-15.

Cox MJ, et al., *Trans R Soc Trop Med Hyg* 1994; 88: 191-97.

Darlow B et al., *Am J Trop Med Hyg* 1982; 31: 1‑9.

Darlow B et al, *Lancet* 1980; 2:1243.

Dulay IS et al., *Papua New Guinea Med J* 1987; 30: 281-290.

Freerksen E et al., Chemotherapy 1995 ; 41 : 396-398.

Freerksen E et al., Chemotherapy 1996 ; 42 :391-401.

Genton B et al., *Ann Trop Med Parasitol* 1994a; 88: 263-270.

Genton B et al., *Trans R Soc Trop Med Hyg* 1994b; 88: 537-541.

Genton B et al., *Ann Trop Med Parasitol* 1995 ; 89, 359 - 376 and 377 - 380.

Georg et al., *Chemotherapy*, in press.

Grimmond T et al., *Papua New Guinea Med J* 1976;19:184‑185.

Hansford CF, Hoyland J, *S Afr Med J* 1982 ;61 :512-514.

Hutchinson DBA, Farquhar JA, *Rev Inf Dis* 1982 ;4 :419-425.

Jones B et al., *BMJ* 1996 ;313 :36-39.

Mueller-Brundaler U et al., *Chemotherapy* 1997 ;43 :451-452.

Price et al., *Trans R Soc Trop Med Hyg* 1995; 89: 523-527.

Schuurkamp GJT. Thesis. Port Moresby, Papua New Guinea: University of Papua New Guinea, 1992. 350p.

Trenholme K et al., *Trans R Soc Trop Med Hyg* 1993;87:464‑466.

Urbaschek R et al., *Antimicrob Ag Chemother* 1991 ;35 :1666-1668.

WHO Global TB Programme, Geneva, WHO 1997.

Yung A, Bennett N. *Med J Aust* 1976;ii:320‑321.

**12 SIGNATURE PAGE**

Protocol title: Efficacy and tolerance of COTRIFAZID (rifampicin, co-trimoxazole, isoniazid) against resistant malaria in semi-immune subjects: a multicentric, open, block-randomised, comparative trial

We agree

1) to perform the clinical trial as specified and in accordance with Good Clinical Practice.

2) to use the trial material, including drug, ONLY as specified in the protocol.

3) that changes to the protocol must be made in the form of an amendment which has the prior approval of all collaborators.

4) that any violation of the protocol may lead to early termination of the trial.

5) to notify the clinical monitors within one working day of any immediately reportable adverse event or abnormal laboratory test (see section 5.3.3), whether considered treatment-related or not, and whether expected or not.

6) to comply with regulatory authority requirements regarding source data verification and the auditing of this trial.

**Investigator**

Name:

Address:

Signature: Date:

**Co-Principal Investigator**:

Name:

Address:

Signature: Date:
